# Ambient Assistive Technologies (AAT): socio-technology as a powerful tool for facing the inevitable sociodemographic challenges?

**DOI:** 10.1186/1747-5341-5-8

**Published:** 2010-06-07

**Authors:** Astrid M Schülke, Herbert Plischke, Niko B Kohls

**Affiliations:** 1 Generation Research Program, Human Science Center, Ludwig-Maximilians-University Munich, Germany; 2 Peter Schilffarth Institut für Soziotechnologie (PSI), Bad Tölz, Germany; 3 Brain, Mind and Healing Program, Samueli Institute, Washington, USA

## Abstract

Due to the socio-demographic change in most developed western countries, elderly populations have been continuously increasing. Therefore, preventive and assistive systems that allow elderly people to independently live in their own homes as long as possible will become an economical if not ethical necessity. These respective technologies are being developed under the term "Ambient Assistive Technologies" (AAT). The EU-funded AAT-project *Ambient Lighting Assistance for an Ageing Population *(ALADIN) has established the long-term goal to create an adaptive system capable of improving the residential lighting conditions of single living elderly persons also aiming at supporting the preservation of their independence.

Results of an earlier survey revealed that the elderly perceived their current lighting situation as satisfactory, whereas interviewers assessed in-house lighting as too dark and risk-laden. The overall results of ALADIN showed a significant increase in well-being from the baseline final testing with the new adaptive lighting system.

Positive results for wellbeing and life quality suggest that the outcome effects may be attributed to the introduction of technology as well as to social contacts arising from participating in the study. The technological guidance of the study supervisors, in particular, may have produced a strong social reactivity effect that was first observed in the famous Hawthorne experiments in the 1930s. As older adults seem to benefit both from meaningful social contacts as well as assistive technologies, the question arises how assistive technology can be socially embedded to be able to maximize positive health effects. Therefore ethical guidelines for development and use of new assistive technologies for handicapped/older persons have to be developed and should be discussed with regard to their applicability in the context of AAT.

## Introduction

In most developed Western societies two long-term socio-demographic trends are becoming gradually more visible: First, the birth rate is below that which is necessary to retain a sustainable population. Second, individual life expectancy is steadily increasing due to improvements in medical science and health care. Even though the speed and course of demographic change, and social and economic contexts may vary throughout different parts of the world, the aging of the population has already become a global trend in many Western societies that will gradually have profound impact upon the life circumstances of many individuals [1]. Thus, governments will have to find culturally and ethically sustainable ways to deal with an aging and shrinking population and the economic consequences.

According to the Congressional Research Service Report the birth rate in the United States of America fell significantly from 1957 to 2003 [2]. Although the forecasted socio-demographic change is considerable in the United States, it is supposed to be even more severe in other parts of the world. In particular, Europe must deal with the consequences of a massively growing populus of elderly people in the near future [3]. The difference between the United States and Europe as regards this sociodemographic situation can - at least to some degree - be attributed to higher migration rates and also higher birth rates (especially among the Hispanic population of the United States, which may partly help to counterbalance the shrinking and aging other domains of the US-population) [2]. Europe, in contrast neither profits from a high migration rate, nor from population groups with high birth rates that could lead to a slowing of such socio-demographic change. This socio-demographic development in Europe is mirrored by the total birth rate in Germany: while it was at a considerably high(er) level in the 1960s, (both in the German Democratic Republic and the Federal Republic of Germany), it has fallen to a level below replacement rate, and as a consequence, the population has been steadily shrinking since 2003 [4].

Obviously, changes in an aging population will have meaningful impact upon the medical and health care sector [5]. This prompts the question of how governments should deal with the increased need for potential long-term care, taking into account both the economic and ethical aspects that such care engenders. Moreover, cultural facets of this socio-demographic change must be considered, if genuine (ethically sound) attention is to be afforded to context-dependent needs of vulnerable populations such as the handicapped and fragile elderly.

Active Aging and Ambient Assistive Technologies as technological and political strategies to address problems of age-related sociodemographic change

Apparently, there is no facile, straightforward solution for this complex problem. However, to avoid the high cost of health insurance as well as to extend the period of independent and unrestricted living for the elderly population, it is vital to develop preventive techniques and technologies that allow the elderly - even under deteriorating health conditions - to live well and independently as long as possible. The European Union (EU) has focused a research and social innovation agenda upon programs that support what is called "active aging". Basic components of the "active ageing" strategy comprise life-long learning, extension of working life, later and gradual transit to retirement, an active life in retirement, as well as performance and health promoting innovations [6]. As a consequence, the governing body of the EU has established a specific program dedicated to fostering research in the field of "Ambient Assisted Living" (AAL), and "Ambient Assistive Technologies" (AAT) as specific, integral components of AAL [7]. AAL relates to concepts, products and services which combine and improve new technologies and social environment with the aim to increase life quality for people in all periods of life. They can also be understood as assistive systems particularly dedicated for a healthy and independent living of elders. Respective research projects in this context were invited for the first time in the 6^th ^framework program (duration: 2002 - 2006) of the EU http://ec.europa.eu/research/fp6/index_en.cfm together with Information Society Technologies http://cordis.europa.eu/ist/.

AATs are designed to contain potential risks in households, by supporting the elderly in everyday activities. Intelligent technologies for an aging population may thereby be helpful for retaining and/or regaining health, and fostering independence, at least until residential care becomes unavoidable. AATs working with ubiquitous computing apply technology in a way that allows a "cloud of care" concept to be realized. This "cloud of care" has been suggested as a unobstrusive framework for integrating technology and providing services in a "smart" and non-invasive way to support people in the management of everyday activities (including medical services) in their homes [8].

In a recent survey, the major goals of technological systems designed to support older adults were summarized as [9]:

1. Assurance systems described by Pollack are aimed primarily at ensuring safety and well-being and reducing caregiver burden by tracking an elders' behavior(s) and providing real-time status reports (e.g. motion and position sensors).

2. Compensation systems to provide guidance to elderly individuals as they execute daily activities, reminding them of training or exercises and other necessities and how to do it (e.g. alarm-clocks for the intake of pharmaceuticals).

3. Assessment systems to infer a person's health, for example, by assessing cognitive level of function, based on continual observation of performance and/or monitoring of routine activities. These are subsumed by the term "AAL technologies".

Whereas assurance systems are already available as commercial products, compensation systems that actually intervene and assist elderly individuals in accomplishing daily activities mainly exist as research prototypes. According to Pollack [9], the challenge is primarily in the development of assessment systems that can provide continual, naturalistic assessment of the cognitive and affective status of older adults.

There are many venues for conceptualizing, designing and implementing compensation and assessment systems to assist daily activities as well as monitor psycho-physiological condition. Some of the most important environmental factors that affect the psycho-physiological condition of an individual are the surrounding lighting parameters. However, the age-dependency of lighting conditions has only recently become a focal research interest. The project "*Ambient Lighting Assistance for an Ageing Population *- ALADIN" was one of the first research endeavors to access the impact of light with special respect to the elderly population. In the following section, we describe the ALADIN project as an example for illustrating how AAT technologies can be employed to foster and/or retain elderly people's independence, health and quality of life.

### Impact of Light on the Elderly and Reasons for Using Light in AAT

#### Physiological and pathological changes associated with the process of aging

It has recently been shown that the impact of light on the organism, and the reaction to environmental lighting conditions may substantially change with age [10]. There are several functional and structural changes that may occur in the visual system (e.g. - particularly in the eye) as a consequence of aging. These include the reduced potential for adaptation of the visual system in elderly people. As the lens and iris become less flexible with progressing age, the retina receives less light, resulting in reduced retinal luminance and a need for increased light levels [11]. Together with age-related diseases of the visual system, such as glaucoma or cataract, these changes lead to impaired vision and color perception.

Chrono-biologically, increased age incurs a gradual desynchronisation of sleep and wake cycles [10], thereby decreasing sleep quality, and also increasing the risk for specific sleep disorders that may decrease daytime vigilance, and correspondingly, quality of life. The sleep - wake cycle depends upon psychobiological "clocks" to synchronize the circadian rhythm with natural sunlight (or an artificial analogue). A poorly illuminated environment may incur negative physiologic effects and represents a risk factor for the desynchronisation of circadian regulation of the CNS and top-down psychophysiologic effects.

#### Ambient Natural Light: improving cognitive performance and reducing negative affect

Due to both physiological and behavioral changes (e.g. more indoor activity), elders are at risk of being exposed to less natural light. Reduced light may be a risk factor for well-being and should be taken into account, when defining the criteria for indoor conditions for this target group. Therefore it is crucial to investigate the temporal and spatial variables of light that need to be applied to mitigate possible negative health effects, and possibly proactively militate salutogenetic effects [12].

As these examples illustrate, elders may be at an increased risk of suffering from insufficient environmental lighting conditions. Thus, implementing higher standards for the indoor lighting conditions - i.e. higher levels of luminance and reduction of glare - for elderly individuals may be an important factor for improving their well-being. However, light conditions in older people's homes have not been a focus of architectural or building attention, and it may thus be assumed that many senior citizens live under suboptimal indoor lighting conditions. If this is to change, the challenge is to specify the lighting conditions (timing of light, its duration as well as its quantity, spectrum, and spatial distribution) to establish a high quality luminous environment specifically designed for the needs and demands of elderly individuals. In the ALADIN project this challenge was accepted. The focus of this paper lies within the discussion of ethical, social and legal aspects of ambient assistive technologies. Additionally, some considerations concerning AATs will be provided to address the possibility of AAL on a large scale. (A detailed description of the study and its results will be published in a forthcoming issue of *Synesis- A Journal of Science, Technology, Ethics, and Policy)*. Additional information about the project is available online at http://www.ambient-lighting.eu.

## Background and Results of the ALADIN project

The fundamental hypothesis of this study was based on the assumption that the ALADIN prototype can be designed in a way that is able to support residents' well-being and quality of life. The long-term aim of the ALADIN project aspired to creating an adaptive system capable of improving the residential lighting conditions so that it may also support the independence of elderly. Time to market is rated at approximately about 5 to 7 years. The importance of this project lies within the core idea of AAT. These new technologies are to contain potential risks in households, and support the elderly in everyday activities. Therefore the project seeks to a) mitigate risks, b) support independence and c) to reduce costs for the health system by prolonging healthy and independent living.

Although an earlier analysis revealed that the elderly did not perceive any need for a change in their current lighting situation, this, however, was in strong contrast to the assessment of interviewers, who rated in-house lighting conditions as too dark and risk-laden.

The ALADIN prototype was explicitly designed to foster mental (and physical) fitness of elderly individuals by adjusting the lighting to their psycho-physiological needs and demands. Mental and physical fitness, general well-being, quality of life and sleep quality were measured with standard questionnaire instruments as main outcome criteria as determined in prior studies.

The use of AAT incurred an overall increase in measured well-being and from the baseline final testing.

On the Limitations of technology: Implications for the ALADIN prototype

Technological devices are predominantly developed for young(er) target groups. In the special context of AATs, the elderly are explicitly addressed. Therefore new design guidelines that take the special needs and demands of elderly and/or handicapped persons into account, must be applied.

In the development of the ALADIN prototype elderly individuals were involved at all stages in order to maximize intended effects and acceptance. Nevertheless technological restrictions that are related to current state of the art in available technology have limited the project's outcome. Three examples are of note. First, psycho-physiological parameters cannot be captured without producing artefacts (by movements or surrounding conditions). These artefacts must be eliminated as a first step in analyses which, currently, has limitations in real time. Second, there are only few devices available on the market that allow wireless transmission of data from a sensor to a computer with reasonable power consumption. (Note that current available solutions usually working either with infrared or blue-tooth technology are both still quite energy consuming). Third, up to now there have been few, less-complex and easy-to-compute psycho-physiological parameters that can be analysed online. Real time-computation of more sophisticated psycho-physiological parameters, (such as heart rate variability or electroencephalography) are, for the time being, simply too complex and dependent on uncontrollable factors to be used by these types of systems in real time.

## Discussion of social and ethical aspects of AAT

### Social aspects of AAT

The positive impact upon well-being may, at first glance, be interpreted as a success of the project given that well-being is intimately connected to mental health. Nevertheless, such results cannot be attributed to a single factor. Other variables, such as social interaction effects also must be taken into account. Thus, these results may reflect the famous Hawthorne-experiments, in which the effects of social presence and awareness, as well as the effect(s) of significantly impacted outcomes have been observed [13]. This "reactivity" phenomenon - viz "Hawthorne effect" [14]- could also contribute to socially relevant outcomes of any medical technology. Especially in this study, but most probably true for other studies in the field of AAL, test-persons received technological guidance from support staff whenever they asked for it and thus were able to establish a contact with someone who was not part of their familiar social environment.

From this perspective, we must scrutinize the effectiveness of any such assistive technological systems. The costs for these new technologies are considered to be quite high, and this prompts the question of whether social support alone could possibly attain the same or even better results. However, given that AATs (and other technologies) are not likely to be used as technological stand-alone-solutions, then it is important to consider the dynamic interaction of technologic expectation within a social milieu, and if any effects are enhanced when embedding new devices within "Ambient Assisted Living - AAL" systems that afford meaningful social constructs. This however requires a conclusive research on the requests and needs of the elderly as well as on the willingness of the elderly to keep contact or build up new contacts with the help of AAT. Berkman and Syme (1979) have shown that lack of social integration negatively affects morbidity and mortality [14]. Perhaps then, a goal of any such technology aimed at enhancing well-being and quality of life, of both the elderly and young(er) persons (or patients), is not to foster solipsistic independence, but to promote and sustain the ability for interpersonal interaction and connectedness.

### AAT and AAL in the context of health promotion and sense of coherence

Thus, AAT, like any new technology must be framed in a broader, non-technological context to assess both its practical usefulness, as well as its ethical soundness. In 1948 the World Health Organisation (WHO) developed a definition of health as:

*"... a state of complete physical, mental and social well-being and not merely the absence of disease or infirmity." *[15]

This definition was expanded by incorporating the concept of salutogenesis, developed by American-Israeli sociologist Aaron Antonovsky (1923 - 1994). In short, Antonovsky argued that the experience of well-being is associated with a Sense of Coherence (SOC). Antonovsky defined SOC as:

*"...a global orientation that expresses the extent to which one has a pervasive, enduring though dynamic feeling of confidence that one's internal and external environments are predictable and that there is a high probability that things will work out as well as can reasonably be expected." *[16].

SOC was considered to be comprised of three following core subcomponents [17]:

A) Comprehensibility: The perception of the world as being structured, predictable, and explicable that allows an individual to understand his or her environment.

B) Manageability: The conviction that resources that are necessary for an individual in order to meet the demands and requirements are available or can be made accessible.

C) Meaningfulness: The profound experience of the individual life as being meaningful.

This definition was later elaborated in the Ottawa-Charta (1986) to include the personal resources of the individual [18]. The move from a "disease-curative" mindset to one of health promotion programs was influenced by the salutogenic concept:

*"...Health is... a resource for everyday life, not the objective of living. [Health is] ... a positive concept emphasizing social and personal resources, as well as physical capacities ..." *[19]

It is important to bear these definitions of health in mind when evaluating the practical and ethical aspects of technological solutions in the context of assisted living. From our perspective AAL technologies may be best regarded as a technological "tool" as part of a larger set of techniques and strategies toward promoting health concepts. If such strategies are meant to preserve or obtain health, they should not only focus on technological support, but also enable independent living and self determination to facilitate meaningful social involvement. It is noteworthy to recall that social support comprises perceived obtained support and meaningful social integration [20]. Therefore, new technologies should be engaged to both improve aspects of cognitive and physical capability and perhaps develop and maintain meaningful social contacts [21].

### Ethical guidelines for the assessment of ambient assistive technologies

AATs aim at creating a "cloud of care" by means of technological devices that are able to adapt to the psychophysiological needs and demands of individuals. Thus, an important question is whether the elderly or people in general can accept constant monitoring by various sensors. According to our studies, the main concern is of "big brother" scenarios in which physiological data might be usurped or misused. These fears must be seriously considered and thus any such technology must be regarded in light of a meaningful balance between privacy and protection. In this latter case, data relevant to monitor health status could be transferred to a medical center, respective general practitioner, or even public health agency to provide an opportunity to promote and ensure health care. In each case, this information is intended to alert and engage contact with medical personnel. In other words, technology is intended to facilitate inter-personal contact, not merely replace it. However, there is a defined need to acknowledge the trend for technology to be used as both means and end. Often, it is this stand-alone possibility that is used to justify the value of costs incurred in research, development, testing, and evaluation. Given what Lendk referred to as the "Technologic Imperative"- how can, or perhaps should we regard the utility and use of AAL- or any such technology- in light of an obligation to engage such techniques and devices in ways that are morally sound?

It is at this point, where the discussion comes to ethics concerning technology. Therefore we have to differentiate between ethics in form and content. The content based or material ethics follows a hierarchy of values which should serve as adjustment factors for consideration in conflicting situations [22].

The hierarchy of values according to material ethics reflect contemporary Principlism as advocated by Beauchamp and Childress and include [23]:

1. Principle of non-harm

No harm shall be done to anyone using technology

2. Principle of autonomy (personal identity and self-determination)

Use of technology is in accordance with the wishes, ambitions and values of users regardless of how comprehensible these might seem.

3. Principle of welfare (provision principle)

Demands the maximization of possible advantages, minimizes disadvantages and improves the situation of others.

4. Principle of equality (i.e.- justice)

Dictates at least a formal prohibition of irrelevant differentiation, and fair provision of resource(s).

Here however, it is important to consider the more detailed distinction of commutative justice (i.e. - unequal provision of resources based upon unequal need) and distributive justice (i.e. - maximizing good(s) through differential provision of limited resources to a greater number of potential recipients). If we consider such a material, Principalist ethics reflects and may be relevant and applied to AAL (or related technologies), it becomes important to note the somewhat Janusian face of Science and Technology [24]. On one hand science and technology can be viewed as providing means to achieve good(s) and facilitate positive ends in life. On the other hand, is the distinct possibility that this same trajectory will incur negative consequences on individual and/or public levels, based upon the lability of personal and societal values and meanings, and the intended and unintended impact that technology can incur.

Thus, we hold that content based ethics should provide a foundation for guidelines during the development and evaluation of supporting technologies. This is especially important in providing a "cloud of care" to explicitly assess the degree of autonomy, self-determination, freedom of decision and dignity that will be affected by technology. However, we also maintain that technologic development may prompt a re-examination of the moral context and social integrity of ethical principles. So for example, we may question if the person utilizing a distinct technology is still free to decide what to do and what not to do? Does the person have control over technology or does technology have control over the person? To what extent will well-being be improved or perhaps defined? Is the assistive technology affordable and available for all or will this technology incur distinct socio-economic bias? And finally does the technology enhance the comprehensibility, manageability and meaningfulness of life, or simply provide it? Such questions must be addressed, examined, and answered prudently if the developing field of AAL is to maintain both practical utility and remain ethically justifiable.

## Abbreviations

AAL: Ambient Assisted Living; AAT: Ambient Assisted Technologies; ALADIN: Ambient Lighting Assistance for an Ageing Population; CNS: Central nervous system; EU: European Union; SOC: Sense of Coherence; WHO: World Health Organisation.

## Competing interests

The authors declare that they have no competing interests.

## Authors' contributions

AMS participated in the design and realization of the study and drafted the manuscript. NBK helped to draft the manuscript and a project report. HP coordinated the study. All authors read and approved the final manuscript.
